# Endobronchial Chondroma: A Rare Case of Benign Tumor With Atypical High Standardized Uptake Value

**DOI:** 10.7759/cureus.24800

**Published:** 2022-05-07

**Authors:** Marc Assaad, Khalil El Gharib, Ali Kassem, Hussein Rabah, Dany El-Sayegh

**Affiliations:** 1 Internal Medicine, Staten Island University Hospital, Staten Island, USA; 2 Internal Medicine, Northwell Health, Staten Island, USA; 3 Pulmonary Critical Care, Staten Island University Hospital, Staten Island, USA

**Keywords:** cartilage [c26.411], chondrosarcoma, recurrent pneumonia, standardized uptake value, sleeve lobectomy, chondroma

## Abstract

Chondroma is a rare benign tumor of the cartilage and occurs in the tracheobronchial tree, either isolated or as part of the Carney triad. It has been sparsely described in the literature, and some were mislabeled as hamartomas. We herein report a case of a 63-year-old female who was initially treated for community-acquired right middle lobe pneumonia. However, the patient's symptoms persisted warranting further workup, which confirmed the diagnosis of post-obstructive pneumonia. Whole-body positron emission tomography (PET) scan showed a hypermetabolic soft tissue lesion within the middle lobe bronchus, with a standardized uptake value (SUV) of 5.4, which is highly concerning for a primary or a secondary lesion. Since no distant lesions were identified, the patient underwent bronchial sleeve lobectomy of the right middle lobe under the assumption of localized disease. Pathology revealed chondroma, which had an unexpectedly high SUV on the PET scan; follow-up imaging denied any recurrence. Our case presents a rare entity of bronchial tumors with high SUV that presented with post-obstructive pneumonia. The patient's consent for writing this report was obtained.

## Introduction

Chondroma is a rare benign cartilaginous tumor that can occur in the tracheobronchial tree, either isolated or as part of the Carney triad [1]. This entity is sparsely described in the literature, and some cases are misidentified as hamartomas [1,2]. We herein report a case of a patient with an endobronchial chondroma, whose diagnosis was the result of the unique clinicopathological and radiological features of this tumor.

## Case presentation

A 63-year-old female patient, with a smoking history of 50 pack-year, with no other relevant past medical history, presented to the emergency department (ED) with dyspnea on exertion that had progressed to rest, along with right-sided, pleuritic, non-radiating chest pain. The patient also reported a productive cough with purulent blood-streaked sputum, as well as subjective fevers of one-week duration. The patient denied palpitations, recent travel, contact with people with similar symptoms, or other constitutional symptoms. The physical exam was notable for decreased breath sounds at the right lower lung field, associated with fine crackles; the rest of the exam was noncontributory.

Initial blood work was significant for leukocytosis with neutrophilic predominance along with a chest X-ray that revealed bilateral basilar infiltrates. Computed tomography (CT) scan of the chest showed right middle lobe consolidation with an air bronchogram, suggestive of pneumonia, accompanied by axillary, paratracheal, and carinal lymphadenopathies of 1-1.2 cm, the whole clinical picture being suggestive of bacterial pneumonia. The patient was admitted to the hospital and received ceftriaxone for the treatment of presumed community-acquired pneumonia. After completing the antibiotic course, the patient underwent cardiac catheterization in the light of an outpatient positive stress test, which revealed moderate stenosis of the left circumflex artery and more pronounced stenosis of the right coronary artery (RCA). A drug-eluting stent was placed in the RCA, and the patient was discharged from the hospital two days after being admitted with dual antiplatelet agents, atorvastatin, and levofloxacin for five additional days to complete the antibiotic course.

**Figure 1:**
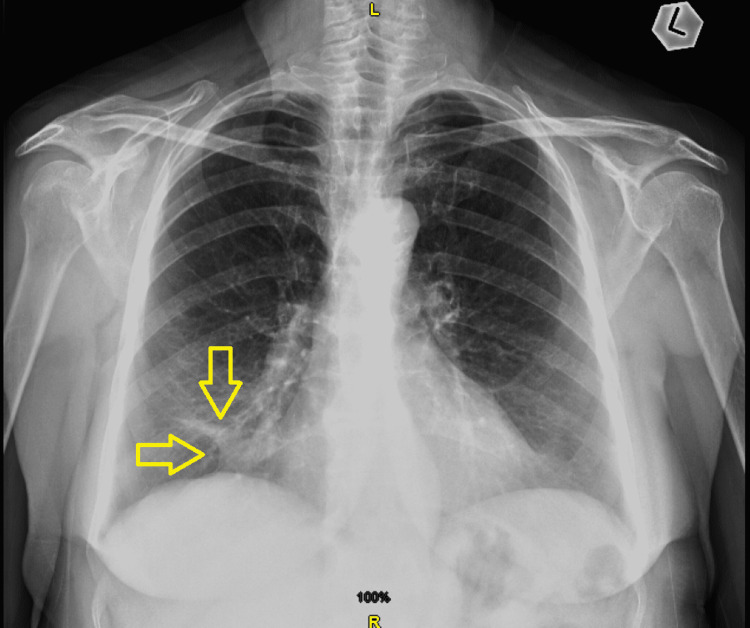
Chest X-ray (posteroanterior view) showing right pericardial triangular opacity of 2.6 x 0.9 cm with atelectatic bands

**Figure 2:**
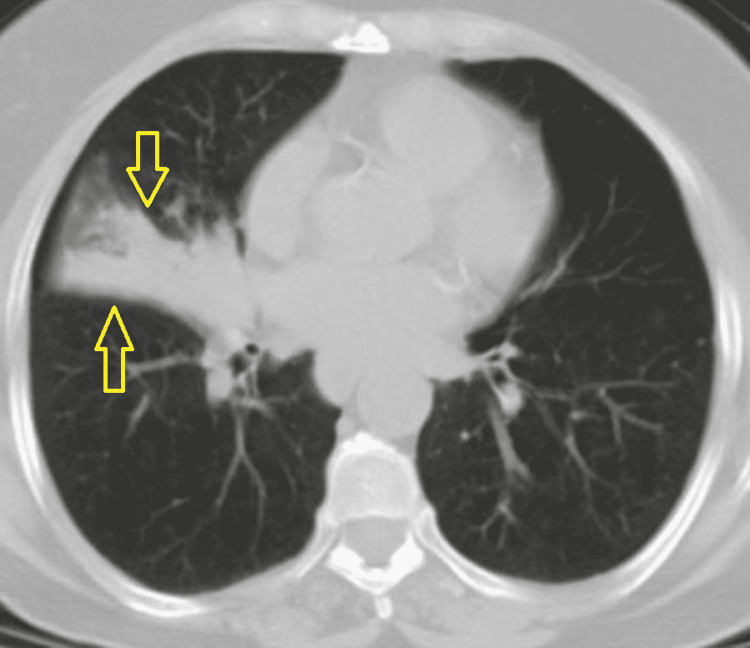
CT scan of the chest showing right middle lobe consolidation

**Figure 3:**
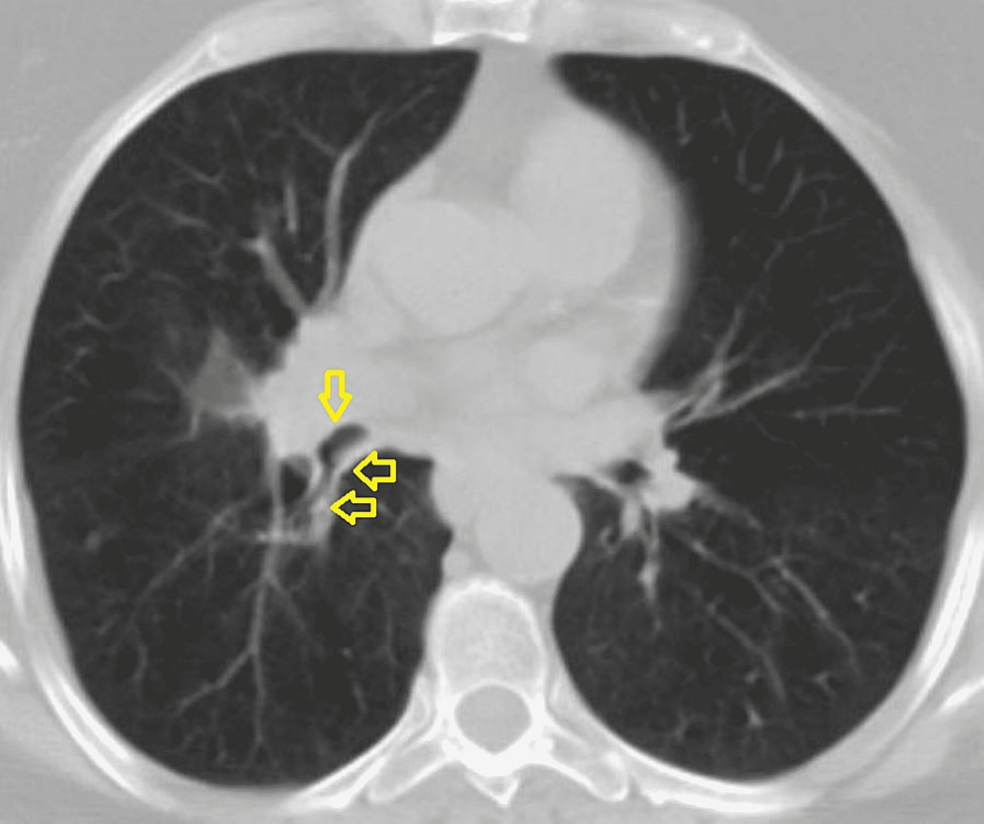
CT scan of the chest showing right middle bronchus obstruction

However, six weeks later, the patient reported only mild symptomatic improvement, and another chest X-ray showed right pericardial triangular opacity of 2.6 x 0.9 cm with atelectatic bands (Figure 1). A repeated CT scan demonstrated an increase in right middle lobe consolidation, along with obstruction of the right middle bronchus (Figures 2, 3).

Because of the latter findings, a bronchoscopy was performed; it documented notable mucosal erythema of the right bronchial tree, with complete obstruction of the right middle bronchus by a solid friable, highly vascularized, and non-calcified endobronchial mass. Minimal contact with the mass resulted in bleeding since the patient was taking dual antiplatelet agents, which were not discontinued. Consequently, no biopsies were taken, and local cytology failed to reveal any atypical cells.

Whole-body fluorodeoxyglucose (FDG) positron emission tomography (PET) was obtained to complete the diagnostic workup, showing hypermetabolic soft tissue lesion within the middle lobe bronchus with a standardized uptake value (SUV) of 5.4, measuring up to 1.5 cm, concerning for a primary lung neoplasm or a carcinoid tumor; no avid intrathoracic adenopathy or distant metastases were found.

**Figure 4:**
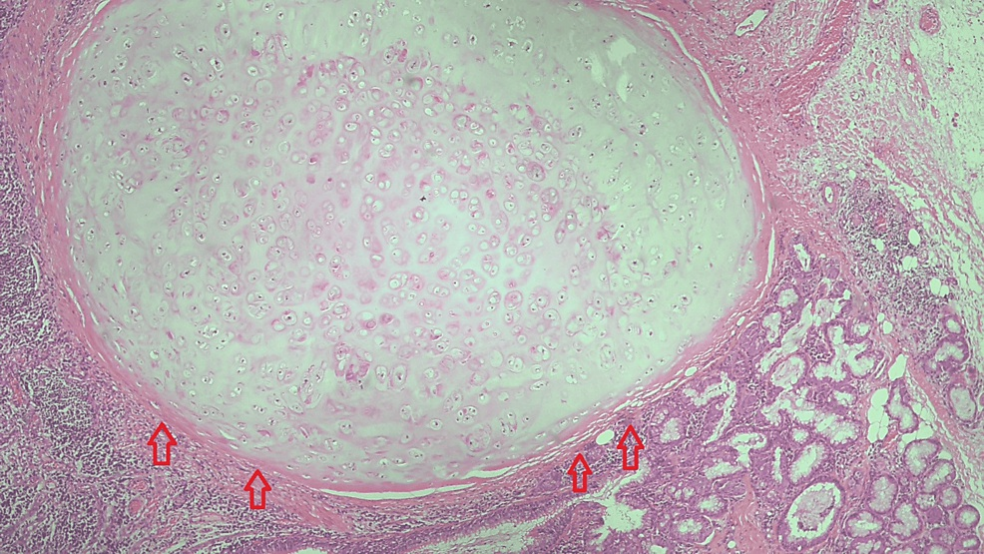
Pathology section of the mass showing lobules of mature and disordered hyaline cartilage, in favor of chondroma

Six months after her stent placement, clopidogrel was stopped and aspirin was continued, and the patient was scheduled for bronchial sleeve lobectomy of the right middle lobe, as the disease remained local, without evidence of regional or distant spread. Exploratory anterolateral thoracotomy of the right hemithorax revealed collapsed right middle lobe that was successfully resected. Pathological examination of the resected mass revealed, as shown in Figure 4, lobules of disorganized, mature cartilage, surrounded by granulation tissue and overlying immature squamous metaplasia, all of which are suggestive of a simple chondroma. Follow-up CT scans of the chest done at three and six months post-lobectomy, and a bronchoscopy done at six months, did not disclose any recurrence, warranting spaced follow-up.

## Discussion

Benign tumors of the tracheobronchial tree are rare; they constitute 0.2% of all endobronchial masses [1], and chondromas, as described by Gaissert and Mark [3], are particularly uncommon. Endobronchial lesions present with endobronchial symptoms like cough, hemoptysis, and complications like obstructive collapse and recurrent pneumonia [4]. Histological examination remains the most important tool to establish the diagnosis and to differentiate whether the tumor is a chondroma or a sarcoma, based on cellularity and atypia, or the presence of bizarre morphology of the cells. The tumor described over here is histologically typical of a benign chondroma, with no features of local invasion, regional spread, secondary distant lesions, and absence of recurrence on regular surveillance. However, it exhibits a radiographic characteristic that might be non-specific, which is the intensity of uptake on PET scans.

Brenner et al. suggested that cartilage usually possesses characteristics such as small cellularity, low rate of mitosis, high quantities of the chondroid matrix, and inactive extracellular matrix, as well as others that render chondroma less amenable to considerable radioisotope uptake [5]. Based on the literature, it is possible to differentiate benign lesions with low SUV max from malignant lesions with high SUV max [6]. Jesus-Garcia et al. defended in their study that a cutoff of 2-2.2 is generally enough to help distinguish chondroma from its sarcomatous counterpart; however, four out of 36 chondromas were found to have an SUV above 2.2 [6]. A systemic review that identified 101 patients from six different studies demonstrated that there is an overlap between benign and malignant lesions, when SUV max ranges between 2 and 4, acknowledging the effect modification bias that some variables introduce such as body habitus, blood pressure, and glycemia.

In this case, we proceeded with sleeve lobectomy. This was described as a successful technique in the management of endobronchial chondromas [7], among other options such as yttrium aluminum garnet (YAG) laser, video-assisted thoracoscopic surgery, and reported cryotherapy [8]. However, viewing that the lesion was found to be benign, the endobronchial approach could have been a possible alternative for lobectomy and thoracotomy.

## Conclusions

Endobronchial chondroma is an unusual benign tumor of the bronchial tree and should be distinguished from well-differentiated chondrosarcoma on pathology. Management of this benign lesion can be limited to endobronchial technique; however, malignant lesions might warrant endobronchial ultrasound staging, lobectomy, and possible adjuvant treatment. PET scan findings in our case did not correlate with pathology findings, thus other imaging or diagnostic tools should be thought to help us differentiate between these entities and guide the therapeutic approach. Hopefully, in the future, more data will be available for better understanding and optimal management of this exceptional cartilaginous tumor.
